# Lipidomic analysis of urinary exosomes from hereditary α‐tryptasemia patients and healthy volunteers

**DOI:** 10.1096/fba.2019-00030

**Published:** 2019-09-30

**Authors:** Sarah C. Glover, Mohammad‐Zaman Nouri, Kubra M. Tuna, Lybil B. Mendoza Alvarez, Lisa K. Ryan, James F. Shirley, Ying Tang, Nancy D. Denslow, Abdel A. Alli

**Affiliations:** ^1^ Division of Gastroenterology, Hepatology and Nutrition College of Medicine University of Florida Gainesville FL USA; ^2^ Department of Physiological Sciences and Center for Environmental and Human Toxicology University of Florida Gainesville Florida; ^3^ Department of Physiology and Functional Genomics and Department of Medicine Division of Nephrology Hypertension, and Renal Transplantation College of Medicine University of Florida Gainesville Florida; ^4^ Department of Pediatrics Pediatric Gastroenterology University of Florida Gainesville Florida

**Keywords:** glycerolipids, glycerophospholipids, hereditary α‐tryptasemia, lipidomics, sterols, urinary exosomes

## Abstract

Exosomes are nano‐sized vesicles that are involved in various biological processes including cell differentiation, proliferation, signaling, and intercellular communication. Urinary exosomes were isolated from a cohort of hereditary α‐tryptasemia (HαT) patients and from healthy volunteers. There was a greater number of exosomes isolated from the urine in the HαT group compared to the control volunteers. Here, we investigated the differences in both lipid classes and lipid species within urinary exosomes of the two groups. Lipids were extracted from urinary exosomes and subjected to liquid chromatography mass spectrometry using a targeted approach. Various molecular species of glycerophospholipids, glycerolipids, and sterols were significantly reduced in HαT patients. Out of a possible 1127 lipids, 521 lipid species were detected, and relative quantities were calculated. Sixty‐four lipids were significantly reduced in urinary exosomes of HαT patients compared to controls. All significantly reduced sphingolipids and most of the phospholipids were saturated or mono‐unsaturated lipids. These results suggest exosome secretion is augmented in HαT patients and the lipids within these exosomes may be involved in various biological processes. The unique lipid composition of urinary exosomes from HαT patients will contribute to our understanding of the biochemistry of this disease.

**Abbreviations**
BST: basal serum tryptase
CE: d7‐cholesterol ester
CER: ceramide
DAG: d7‐diacylglycerol (DAG)
DCER: dihydroceramide
EDTA: ethylenediaminetetraacetic acid
ELISA: enzyme‐linked immunosorbent assay
FD: functional dyspepsia
FGIDs: functional gastrointestinal disorders
HCER: hexosylceramide
HαT: hereditary α‐tryptasemia
IBS: irritable bowel syndrome
LCER: lactosylceramide
LPC: lysophosphatidylcholine
LPE: d7‐lysophosphatidylethanolamine
LPG: lysophosphatidylglycerol
LPI: lysophosphatidylinositol
LPS: lysophosphatidylserine
MAG: d7‐monoacylglycerol
mTORC2: mammalian Target of Rapamycin Complex 2
PA: d7‐phosphatidic acid
PAR2: proteinase‐activated receptor‐2
PC: d7‐phosphatidylchloline
PE: d7‐phosphatidylethanolamine
PG: d7‐phosphatidylglycerol
PGD2: prostaglandin D2
PGE2: Prostaglandin E2
PGI2: Prostaglandin I2
PI: d7‐phosphatidylinositol
PIP2: Phosphatidylinositol 4,5‐bisphosphate
PLA2: phospholipase A2
PLC2: phospholipase C‐2
PS: d7‐phosphatidylserine
RIPA: radioimmunoprecipitation assay buffer
SM: sphingomyelin
SMC: systemic mastocytosis
TAG: d7‐triacylglycerol

## 1 INTRODUCTION

Hereditary α‐tryptasemia (HαT) is a recently identified, genetic disorder associated with elevated basal serum tryptase (BST) and multisystem, clinical phenotypes.^1^, ^2^, ^3^, ^4^ HαT is characterized by dominant inheritance of multiple copies of α‐tryptase‐encoding sequence for *TPSAB1* leading to BST elevations.^1^, ^2^, ^3^, ^4^ The incidence of this genetic disorder is the subject of ongoing investigations. However, elevated BST, which is shared by many of the HαT clinical phenotypes,^4^, ^5^, ^6^, ^7^, ^8^, ^9^ is a relatively common biochemical trait present in 4%‐6% of the general population.^2^, ^4^ Moreover, increasing evidence suggests that HαT is also relatively common, and frequently underlies elevated BST.^1^, ^2^, ^3^, ^4^ Affected individuals exhibit several comorbid functional symptoms including: functional gastrointestinal disorders (FGIDs), atopy, anaphylaxis, arthralgia, connective tissue abnormalities, dysautonomia, and anxiety/depression.^1^, ^3^, ^4^ Among these, FGIDs, including functional dyspepsia (FD) and irritable bowel syndrome (IBS), are frequently reported among HαT patients in the cohorts studied thus far.^1^, ^4^ Indeed, IBS, defined using the Rome III criteria,^10^ was present in half of patients from highly symptomatic families, and in a third of individuals in unselected cohorts 4; approximately 2 to 5 times more prevalent than estimations for North American populations.^11^ Nevertheless, the relevance of increased BST in HαT patients, and the mechanism by which increased α‐tryptase copy number produces the associated multisystem disorder, is not known.

Exosomes are nano‐sized vesicles derived from the endosomal network that have been shown to play an important role in various biological processes including intercellular communication,^12^, ^13^, ^14^, ^15^ cell differentiation,^16^, ^17^ proliferation,^18^, ^19^ and intracellular signaling.^20^ The physiological and pathophysiological roles of exosomes are mediated by the packaged material that they carry which include mRNAs, miRNAs, lipids, and various types of proteins.^21^ Exosomes are secreted by most cell types and can be found in biological fluids including urine, blood, cerebrospinal fluid, and saliva.^22^, ^23^ Exosomes are secreted into the extracellular milieu by exocytosis from multivesicular bodies that originate from late endosomes. Although there is a clear understanding of exosome biogenesis, the mechanism(s) for the uptake of exosomes by recipient cells is largely unknown. Potential mechanisms of exosome uptake by recipient cells have been reviewed by McKelvey et al^24^ These mechanisms include receptor‐mediated endocytosis, raft‐mediated endocytosis, fusion, macropinocytosis, phagocytosis, and juxtacrine signaling.^24^ The mechanism of exosome formation suggests the inner and outer lipid layers of exosomal membranes have the same orientation as the plasma membrane of the cells from which they originate. Importantly, exosomes present in biological fluids, such as urine, are a mixed population that originate from various cell types. Dang et al compared the content of two types of exosomes isolated from the conditioned media of mouse cortical collecting duct cells.^25^

In that study, there were significant differences in lipid classes and fatty acid composition of exosomes isolated from conditioned media collected on the apical side of the cells compared to exosomes isolated from conditioned media collected on the basolateral side of the same cells. Exosomes are enriched in sphingolipids, phospholipids, and cholesterols. Trajkovic et al showed the sphingolipid ceramide (CER) plays an important role in the secretion of exosomes into the extracellular milieu.^26^ This group showed exosome release is attenuated after inhibition of the sphingomyelin (SM) hydrolyzing enzyme neutral sphingomyelinase.^26^ Obata et al showed cellular CER levels decreased when adiponectin mediated increase in exosome biogenesis and secretion occurs.^27^

Urinary lipidomics, in general, has become an attractive area in biomedical sciences since the urine is a rich source of non‐invasive biomarkers and it can be used to investigate disease mechanisms. Lipidomics of urinary exosomes may offer an additional advantage because the packaged cargo within these urinary exosomes may be associated with alterations in various cellular processes including signaling, differentiation, and communication. The aim of this study was to investigate lipid profiles from urinary exosomes of HαT patients compared to healthy volunteers. The results from this study suggest a potential use of exosomal lipids in the diagnosis and characterization of HαT.

## 2 MATERIALS AND METHODS

### 2.1 Human subjects

Urine samples were collected from HαT patients and healthy volunteers for exosome isolation under IRB protocols 201601218 and 201900980. Duodenal biopsies from systemic mastocytosis and HαT patients were obtained under IRB protocol 201702274 in order to make formalin fixed paraffin embedded slides. Patients had either confirmed SMC by bone marrow or confirmed HαT based on a cheek swab revealing duplication or triplication in the tryptase gene (Gene by Gene).^4^ Potential confounding variables between the HαT and the control group were considered and are listed in Table 1. Each HαT patient in our cohort was evaluated for specific clinical characteristics of the disease, which is given in Table 2.

**Table 1:** Potential confounding variables between the control (CTRL) and hereditary α‐tryptasemia (HαT) groups

Variable	Mean ± SEM
Control	
Age (y)	41.6 ± 5.0
Weight (lbs)*	178.2 ± 12.1
Urine volume collected	52.5 ± 5.6
HαT	
Age (y)	30.5 ± 6.3
Weight (lbs)*	123.4 ± 16.0
Urine volume collected (mL)	45.2 ± 4.9

* There is a statistically significant difference between the two groups for weight.

**Table 2:** Twenty‐one (18 female and 3 male) patients with HαT evaluated for the highest measured serum tryptase level, mast cell count in the gastrointestinal tract, existence of gastrointestinal dysmotility symptoms (Y = yes, N = no), existence of inflammation in the gut, and alpha and beta copy numbers of tryptase gene. Eleven patients from our cohort (n = 21) were randomly selected for isolating exosomes from the urine. Urinary exosomes were isolated from urine specimens collected between 9‐11 AM

Sample	Max tryptase	Mast cells	GI dysmotility	Gut inflammation	Alpha copy number	Beta copy number
Female 1	9.2	47	Y	Eosinophilic gastroenteritis	3	2
Female 2	12	ND	Y	N	2	3
Female 3	12.8	28	Y	N	2	3
Female 4	30	65	Y	N	2	3
Female 5	13.4	ND	N	Crohn's	3	2
Female 6	14.6	25	N	Microscopic colitis	2	3
Female 7	13	54	Y	N	3	2
Female 8	22	72	Y	N	2	3
Male 1	11	ND	ND	ND	2	3
Female 9	21	45	Y	N	2	3
Female 10	13.4	35	Y	N	3	2
Female 11	8.1	23	Y	N	3	2
Female 12	9.9	42	N	N	3	2
Female 13	13.1	59	Y	Y	2	3
Female 14	13.1	ND	Y	Autoimmune gastritis	3	2
Male 2	14.7	ND	Y	N	2	3
Female 15	17.3	ND	ND	ND	4	2
Female 16	15	52	Y	N	3	2
Male 3	26.5	NA	N	Crohn's	2	3
Female 17	10.5	60	N	Crohn's	3	2
Female 18	23	102	Y	Y	2	3

### 2.2 Immunohistochemistry

Immunohistochemistry was carried out by the Molecular Pathology Core at University of Florida. Briefly, 4 μm serial sections were de‐paraffinized, and were treated by Ethylenediaminetetraacetic acid (EDTA) (Richard Allan Scientific Co.) at 98°C for 20 minutes, then cool down on the bench for 20 minutes. Background Sniper (Biocare Medical) were used to reduce unspecific background staining. Sections were incubated with Rabbit anti‐human CD117 antibody (1:500, Abcam) for 60 minutes. The slides were then incubated with Mach2 goat anti‐rabbit HRP polymer (Biocare Medical), the DAB chromagen (Biocare Medical) and CAT hematoxylin counterstain (Biocare Medical).

To detect mast cell tryptase, sections were deparaffinized and rehydrated. Sections were treated with Avidin/Biotin Blocker (Vector Laboratories) to block endogenous Avidin and Biotin. Then sections were treated with mouse IgG to eliminate endogenous mouse IgG for 1 hour. Sections were incubated with mouse anti‐mast cell tryptase (1:2000, Abcam) for 60 minutes followed by incubation with biotinylated goat anti‐mouse immunoglobulin antibodies (Vector, Burlingame, CA) for 30 minutes. Staining was developed using the ABC‐Elite reagents and chromagen DAB (Vector Laboratories) and hematoxylin counterstains.

### 2.3 Chemicals and reagents

High performance liquid chromatography grade methylene chloride, methanol, isopropanol, acetonitrile, ethanol as well as water were purchased from Fisher Scientific. Ammonium acetate was acquired from Sigma‐Aldrich. SPLASH Lipidomix (Avanti Polar Lipids, Inc) used as internal standards was a mixture of 14 compounds including d7‐phosphatidylchloline (PC) (15:0/18:1); d7‐phosphatidylethanolamine (PE) (15:0/18:1); d7‐phosphatidylserine (PS) (15:0/18:1); d7‐phosphatidylglycerol (PG) (15:0/18:1); d7‐phosphatidylinositol (PI) (15:0/18:1); d7‐phosphatidic acid (PA) (15:0/18:1); d7‐lysophosphatidylcholine (LPC) (18:1); d7‐lysophosphatidylethanolamine (LPE) (18:1); d7‐cholesterol ester (CE) (18:1); d7‐monoacylglycerol (MAG) (18:1); d7‐diacylglycerol (DAG) (15:0‐18:1); d7‐triacylglycerol (TAG) (15:0‐18:1); d9‐SM (18:1‐18:1), and d7‐cholesterol. The internal standard mix was diluted five times with cold methanol before use.

### 2.4 Isolation of urinary exosomes

A total of 40 mL of freshly collected urine from HαT patients or healthy donors was centrifuged at 1000× *g* for 15 minutes at 4°C. The supernatant was then filtered using a 0.2 μm rapid‐flow Nalgene filter (Thermo Fisher Scientific). The filtered supernatant was subjected to ultracentrifugation at 118 000× *g* for 70 minutes at 4°C using a fixed‐angle Ti‐70 rotor (Beckman Coulter, Inc). The pellet containing the exosomes was resuspended in 200 µL of ultra‐pure phosphate buffered saline (PBS) consisting of 1.06 mmol/L potassium phosphate monobasic; 2.97 mmol/L sodium phosphate dibasic and 155 mmol/L NaCl, pH 7.4 and 280‐315 mOsm/kg).

### 2.5 Characterization of urinary exosomes

Exosome concentration and size was determined by nanoparticle tracking analysis using a NanoSight NS300 (Malvern Instruments) as previously described.^13^, ^25^, ^28^ For Western blot analysis, an aliquot of the exosomes was first mixed in an equal volume of radioimmunoprecipitation assay (RIPA) buffer (Thermo Fisher) and then sonicated for two 10 second intervals on ice. Protein concentration was determined using the bicinchoninic acid protein assay (Thermo Fisher). Fifty micrograms of total exosomal protein was subjected to sodium dodecyl sulfate, polyacrylamide gel electrophoresis (SDS‐PAGE), and Western blotting to identify exosomal markers (Tsg101, flotillin‐1, annexin A2, and caveolin‐1).

### 2.6 Lipid extraction

Lipids were extracted from exosomes according to the Bligh and Dyer method^29^ as described previously.^25^ Briefly, 6 µL of each purified exosome sample (Table 3) was mixed with 0.994 mL water, placed in 10‐mL glass screw‐capped tube and kept on ice for 10 minutes. A mixture of 0.9 mL methanol and 2 mL methylene chloride was added followed by vortexing for 30 seconds. Samples were spiked with 50 µL internal standard, vortexed and then incubated at room temperature for 30 min. One mL water and 0.9 mL methylene chloride were added, and samples were gently inverted 10 times and then centrifuged at 200 *g* for 10 minutes. The organic lower phase (methylene chloride) was carefully collected using a glass Pasteur pipette, concentrated to dryness under an N_2_ stream and reconstituted into 50 µL of ethanol before analysis.

**Table 3:** Concentration of exosomes in particles/6μL for each control (CTRL) or hereditary α‐tryptasemia (HαT) sample used for lipidomics

Sample	Concentration (particles/6μL)	Sample	Concentration (particles/6μL)
CTRL1	9.2 × 10^8^	HαT1	9.4 × 10^8^
CTRL2	1.5 × 10^9^	HαT2	1.3 × 10^9^
CTRL3	4.4 × 10^8^	HαT3	3.4 × 10^8^
CTRL4	5 × 10^8^	HαT4	9.6 × 10^8^
CTRL5	6.6 × 10^8^	HαT5	3.2 × 10^9^
CTRL6	1.2 × 10^9^	HαT6	2.2 × 10^9^
CTRL7	8.3 × 10^8^	HαT7	6.7 × 10^8^
CTRL8	1.7 × 10^9^	HαT8	9.6 × 10^8^
CTRL9	2.4 × 10^8^	HαT9	2.3 × 10^9^
CTRL10	2.1 × 10^8^	HαT10	1.2 × 10^9^
CTRL11	5.7 × 10^8^	HαT11	1.8 × 10^9^

### 2.7 LC‐MS/MS conditions

Lipid samples were analyzed using an ultra‐high‐performance liquid chromatography system (UHPLC, Shimadzu Co.) coupled to a QTrap 6500 mass spectrometer (AB Sciex, Redwood Shores). Chromatographic separation was performed using an XBridge Amide 3.5 µm, 4.6 × 150 mm column (Waters, Ireland). A binary gradient was applied using acetonitrile: water with the ratio of 95:5 (v/v) and 50:50 (v/v) for mobile phase A and B, respectively. Both solvents contained 1 mmol/L ammonium acetate and the pH of freshly prepared mobile phases was adjusted to 8.2. The linear gradient of solvent B increased to 6% in 6 minutes, and then reached to 25% within 4 minutes, 98% within 1 minute and finally 100% within 2 minutes with the flow rate of 0.7 mL.min^−1^. After each separation, the column was flushed using mobile phase B at a flow rate of 1.5 mL.min^−1^ for 3 minutes 5 µL of sample was injected to the UHPLC and the effluent was transferred to the ion source using nanoViper fittings and tubing with 100 µmol/L ID (nanoViper capillary IDXL, Thermo Scientific). Mass Spectrometry was operated in scheduled MRM with both negative and positive ion modes (Table 4).

**Table 4:** Lipid categories and number of lipid species that scanned and quantified using targeted mass spectrometry

Lipids (category)	Scan mode	Scanned lipid species	Quantified lipid species	Significantly changed lipid species
Glycerophospholipids				
PC	Negative	79	4	2
PE	Negative	142	32	5
PG	Negative	78	28	8
PI	Negative	77	10	2
PS	Negative	78	4	—
LPC	Negative	16	8	—
LPE	Negative	16	9	8
LPG	Negative	16	—	—
LPI	Negative	16	—	—
LPS	Negative	16	—	—
Sphingolipids				
SM	Positive	12	12	2
CER	Positive	12	12	2
DCER	Positive	12	12	3
HCER	Positive	12	7	—
LCER	Positive	12	—	
Glycerolipids				
TAG	Positive	445	342	13
DAG	Positive	50	17	6
MAG	Positive	17	3	—
Sterols				
CE	Positive	21	21	13
Total		1127	521	64

Abbreviations: CE, cholesterol ester; CER, ceramide; DAG, diacylglycerol; DCER, dihydroceramide; HCER, hexosylceramide; LCER, lactosylceramide; LPC, lysophosphatidylcholine; LPE, lysophosphatidylethanolamine; LPG, lysophosphatidylglycerol; LPI, lysophosphatidylinositol; LPS, lysophosphatidylserine; MAG, monoacylglycerol; PC, phosphatidylcholine; PE, phosphatidylethanolamine; PG, phosphatidylglycerol; PI, phosphatidylinositol; PS, phosphatidylserine; SM, sphingomyelin; TAG, triacylglycerol.

The settings of the electrospray ionization source were as follows: Declustering potential for positive and negative modes was set to 60 and 80, respectively. Collision energy was varied from 25 to 60 in both positive and negative modes depending on the lipid species. Other fixed parameters were entrance potential and collision cell exit potential which were set at 10 and 15, respectively. Ion spray voltage was kept at 4.5 kV and temperature was 300°C. Each sample was injected twice as technical replicates. Cross contamination was avoided using the following three techniques: flushing column and tubing using a high flow rate of mobile phase B, extra washing of needle using 500 µL isopropanol and running blanks as samples throughout the procedure at set intervals.

### 2.8 Lipid quantitation

Data from a total of 19 lipid groups, including PC, PE, PI, PG, PS, LPC, LPE, lysophosphatidylinositol (LPI), lysophosphatidylglycerol (LPG), lysophosphatidylserine (LPS), TAG, DAG, MAG, CE, SM, CER, dihydroceramide (DCER), hexosylceramide (HCER), and lactosylceramide (LCER) were acquired using Analyst software (ver. 1.6.2). All peaks were inspected without Gaussian smoothing and relative amounts of different lipid classes were calculated based on the concentration of the related internal standard using MultiQuant software (ver. 3.0.3). Instrument performance was checked using lipid profile of bovine heart extract as a standard. Values were mean of 11 biological and 2 technical replicates and data were normalized based on the equal exosome number in HαT patients and healthy volunteers. Differences in the relative concentrations of each lipid species were statistically analyzed at the probability levels of 1% and 5% using a t‐test.

## 3 RESULTS

### 3.1 Differences in mast cells in the duodenum of HαT patients

**Figure 1:**
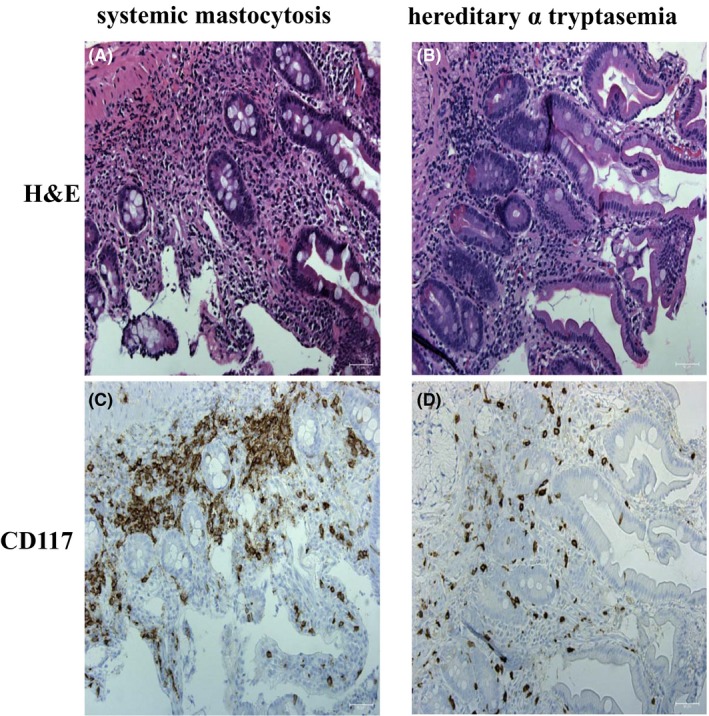
CD117 expression in patients with hereditary alpha tryptasemia compared to systemic mastocytosis. Panel A and C are representative hematoxylin and eosin (H&E) and CD117 stains of duodenum of a patient with systemic mastocytosis. Panel B and D are representative H&E and CD117 from duodenum of a patient with hereditary alpha tryptasemia (HαT). Mast cells are clustered, expanded in number, and spindle shaped in systemic mastocytosis. In HαT, CD117 positive mast cells are expanded in the lamina propria with slightly clustering and are increased in cell size. CD117 positive cells stain brown. Slides stained for CD117 are counterstained with hematoxylin. Bar length = 50 µm

**Figure 2:**
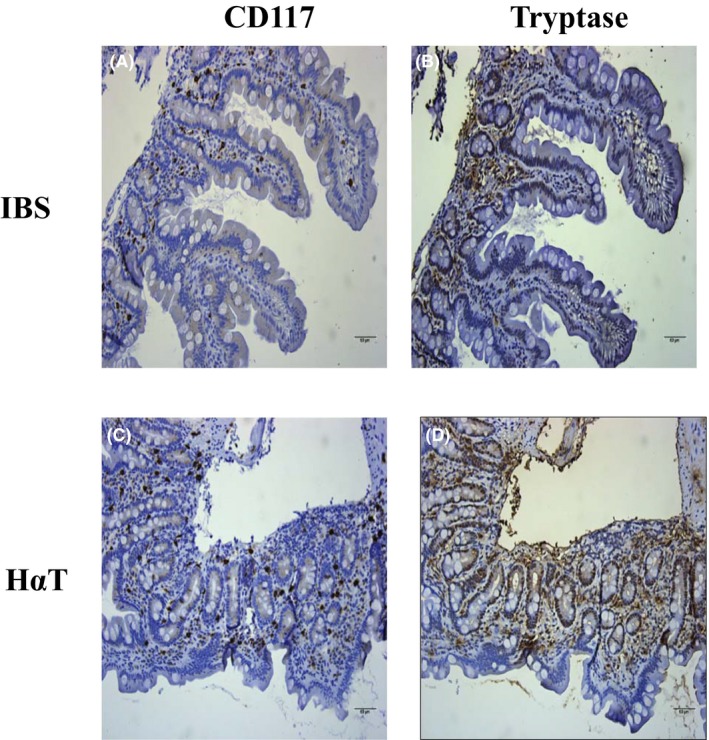
CD117 and tryptase expression in patients with hereditary alpha tryptasemia compared to irritable bowel syndrome. Panel A and B are representative CD117 and tryptase stains of duodenum of a patient with irritable bowel syndrome (IBS). Panel C and D are representative CD117 and tryptase from duodenum of a patient with hereditary alpha tryptasemia (HαT). In contrast with IBS, there are increased numbers of lamina propria mast cells in HαT as well as increased amount of tryptase release into the surrounding tissue. CD117 and tryptase are in brown. Slides are counterstained with hematoxylin. Bar length = 60 µm

Mast cells can be defined by being CD117^+^ and FceR1^+^ Figure 1 shows the distribution and number of mucosal mast cells in the duodenum in systemic mastocytosis and HαT. The mast cells from patients with systemic mastocytosis are increased in number, clustered, and spindle shaped. The mucosal mast cells in HαT also appear to be increased in number but are only minimally clustered and are larger rather than spindle shaped. Figure 2 shows the difference between IBS and HαT in the duodenum by CD117 and tryptase. In HαT, the mast cells are increased in number and appear to express more tryptase.

### 3.2 Increased number of urinary exosomes from HaT patients compared to healthy volunteers

**Figure 3:**
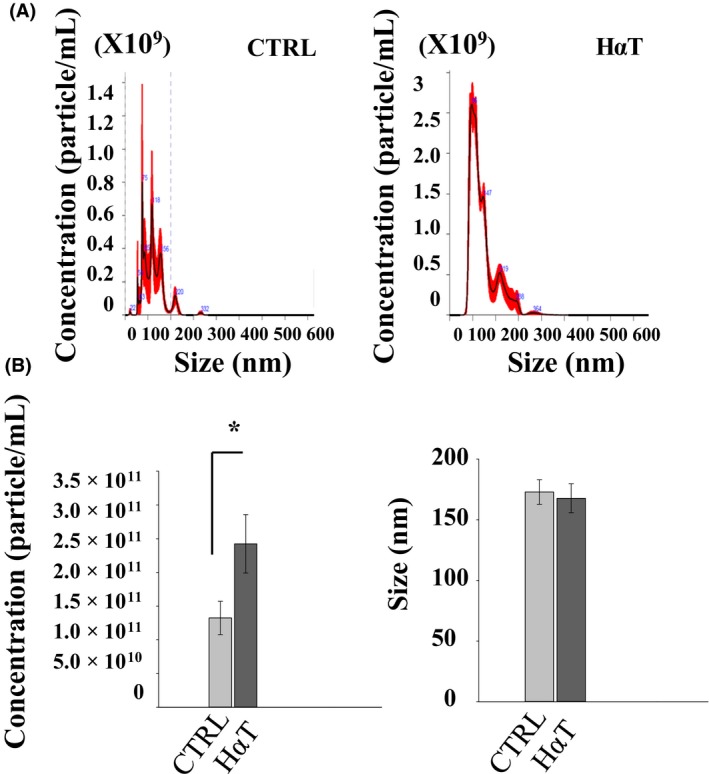
Characterization of urinary exosomes from patients with HαT compared to healthy volunteers. (A), Representative nanoparticle tracking analysis showing concentration and size of the urinary exosomes isolated from HαT patients or healthy volunteers (ctrl). (B), Summary bar graphs of exosome concentration and size from the two groups. *represents statistical significance (*P* < .05)

Urinary exosomes contain a mixed population of exosomes that originate from multiple sources including renal epithelial cells in each segment of the nephron but also from other cell types. Larger exosomes are not thought to be readily filtered due to their size and negative charge, but exosomes may be secreted into the tubule lumen. Here, we show HαT patients have more exosomes in the urine (2.5 × 10^11^) compared to that of healthy volunteers (1.4 × 10^11^) (Figure 3). The mean size of these urinary exosomes was not different between the two groups and measured roughly 170 nm in diameter. Certain variables were considered between the two groups. The control group consisted of healthy volunteers (control group) with an average age (±SEM) of 41.64 ± 5.00 compared to 30.46 ± 6.34 for the HaT group. The average weight (±SEM) was 178.18 ± 12.08 for the control group compared to 123.36 ± 16.03 for the HaT group. The amount of urine collected was 52.46 ± 5.62 for the control group and 45.18 ± 4.87 for the HaT group, represented as mean ± SEM.

### 3.3 Isolation and characterization of urinary exosomes from HαT patients

**Figure 4:**
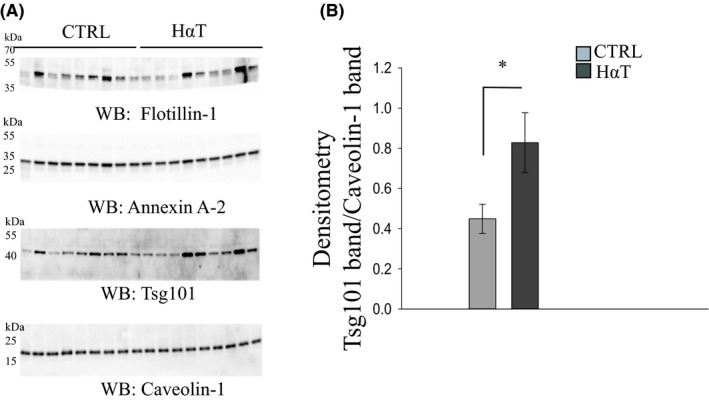
Characterization of urinary exosomes from HαT patients and healthy volunteers by Western blot analysis. Panel A shows Western blots for four extracellular vesicle markers. Thirty micrograms of exosomal lysate was resolved by SDS‐PAGE before the proteins were transferred to nitrocellulose membranes in order to probe for multiple exosomal markers by western blot using specific antibodies against flotillin‐1, annexin A2, Tsg101, and caveolin‐1. There were no observable differences in caveolin‐1 protein expression between control (CTRL) and HαT samples. Panel B shows the densitometry for the Tsg101 Western blot normalized to the caveolin‐1 Western blot. * indicates significance at *P* < .05

Exosomes contain various proteins that originate from the endosomal compartment that are routinely used for characterization of exosomes. Among these exosomal marker proteins, we considered Tsg101, annexin A2, flotillin‐1, and caveolin‐1. We performed Western blotting using polyclonal antibodies against these proteins after resolving the exosomal lysates by SDS‐PAGE to further characterize the nano‐sized vesicles isolated from the urine of HαT patients and healthy volunteers as urinary exosomes (Figure 4A). Expression of the exosome‐specific marker Tsg101^30^, ^31^, ^32^ was greater in urinary exosomes from HαT patients compared to the control group (Figure 4B).

### 3.4 Lipidomics analysis

A total of 1127 lipids were scanned in the employed method, of which 521 lipid species were identified and quantified in HαT patients and healthy volunteers (Table 4). These fell into four broad groups of lipids, including glycerophospholipids, sphingolipids, glycerolipids, and sterols. A total of 64 lipid species (12.2% of the total) were significantly different suggesting that the majority of the phospholipids in the membranes of urinary exosomes of HαT patients and healthy volunteers were similar.

### 3.5 Glycerophospholipids and glycerolipids in urinary exosomes of HαT patients

**Figure 5:**
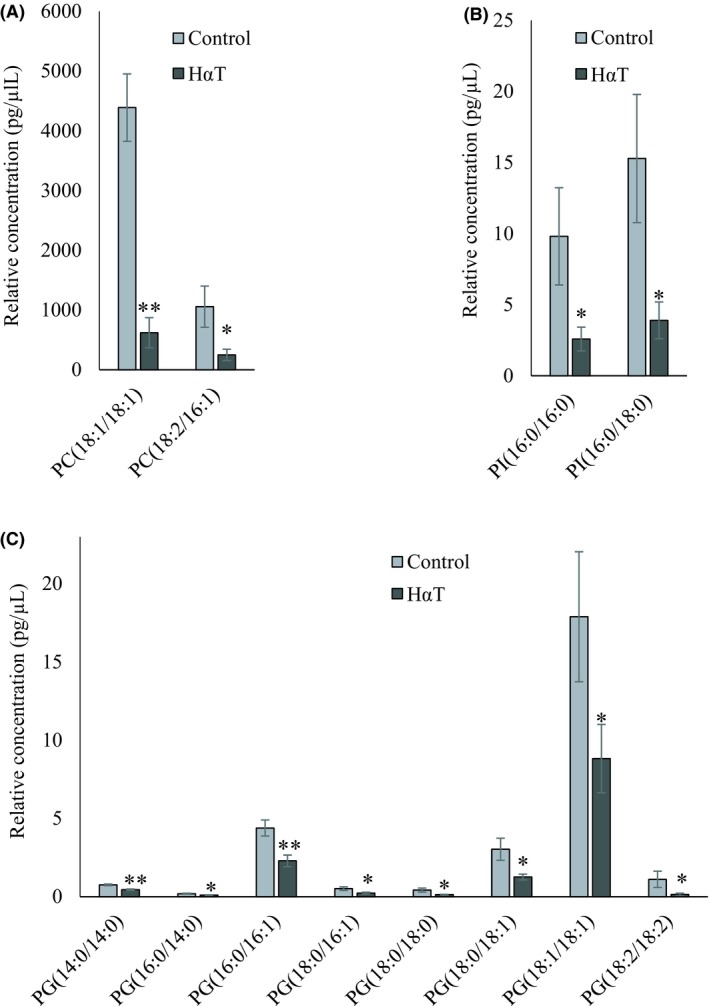
Relative quantity of glycerophospholipids in freshly isolated urinary exosomes from HαT patients and healthy volunteers. Panel A, B, and C represent significantly changed phosphatidylcholine (PC), phosphatidylinositol (PI) and phosphatidylglycerol (PG), respectively. Data are mean ± SE of relative quantity (pg/µL). * and ** indicate significant at 5% and 1% probability levels, respectively

**Figure 6:**
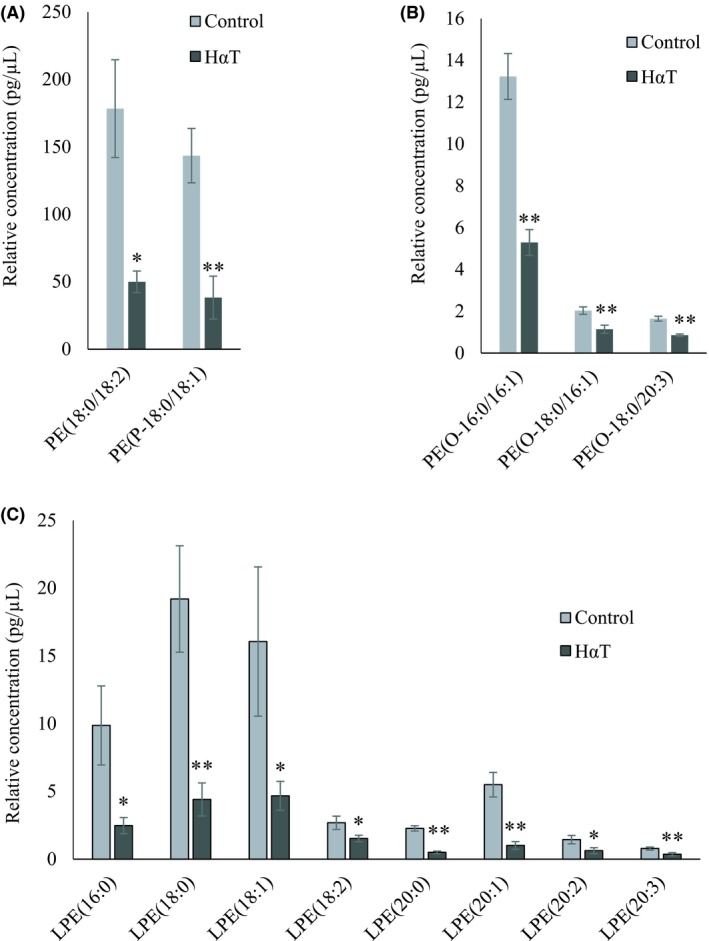
Relative quantity of phosphatidylethanolamines in freshly isolated urinary exosomes from HαT patients and healthy volunteers. Panel A, B and C represent significantly changed phosphatidylethanolamine (PE) and PE‐P, PE‐O and lysophosphatidylethanolamine (LPE), respectively. Data are mean ± SE of relative quantity (pg/µL). * and ** indicate significance at *P* < .05 and < .01, respectively

A total number of 534 glycerophospholipid species were scanned, of which 95 were identified and quantified and 25 were significantly different, roughly 26% of the total. Among the identified glycerophospholipids, the following species were significantly reduced in HαT patients: Two PC, PC(18:1/18:1) and PC(18:2/16:1); five PE, PE(18:0/18:2), PE(P‐18:0/18:1), PE(O‐16:0/16:1), PE(O‐18:0/16:1) and PE(O‐18:0/20:3); eight PG, PG(14:0/14:0), PG(16:0/14:0), PG(16:0/16:1), PG(18:0/16:1), PG(18:0/18:0), PG(18:0/18:1), PG(18:1/18:1), PG(18:2/18:2; two PI including PI(16:0/16:0) and PI(16:0/18:0) and eight LPE with 16‐20 carbons and with 0‐3 double bonds (Figures 5 and 6).

**Figure 7:**
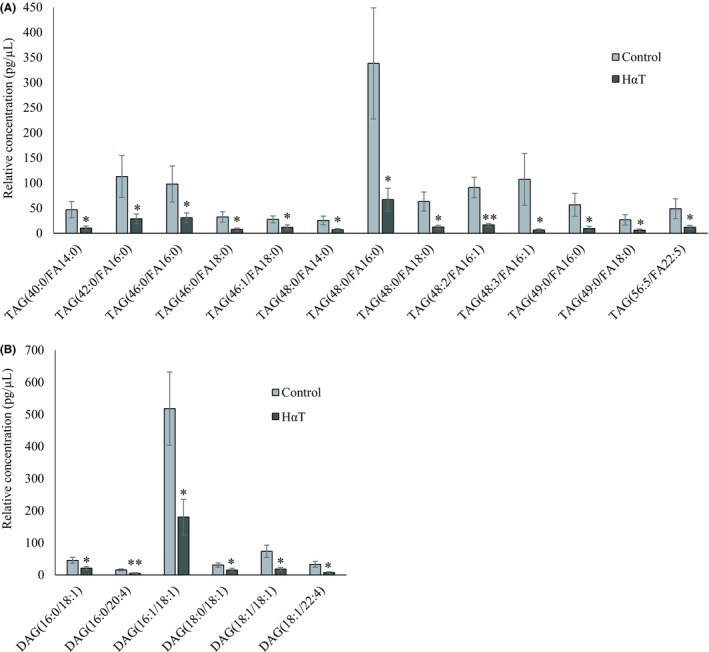
Relative quantity of glycerolipids in freshly isolated urinary exosomes from HαT patients and healthy volunteers. Panel A represents various significantly changed triacylglycerol (TAG) and Panel B, diacylglycerol (DAG) species. Data are mean ± SE of relative quantity (pg/µL), * and ** indicate significance at *P* < .05 and < .01, respectively

Out of 512 glycerolipid species, 342 TAG, 17 DAG, and 3 MAG were identified and quantified in HαT patients and healthy volunteers (Table 4). Thirteen TAG and 6 MAG, together constituting about 5% of this group, were significantly reduced in HαT patients after comparing the quantity of the lipid species in two groups (Figure 7). This suggests that 95% of the lipids in this class remained roughly unchanged in the two exosome groups.

### 3.6 Sphingolipids and sterols in urinary exosomes of HαT patients

**Figure 8:**
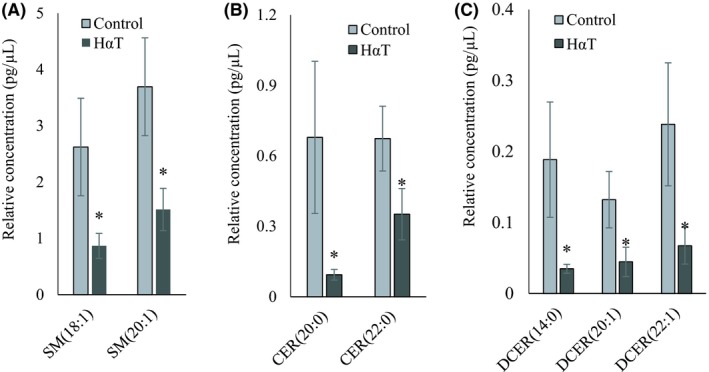
Relative quantity of sphingolipids in freshly isolated urinary exosomes from HαT patients and healthy volunteers. Graphs A, B and C represent Sphingomyelin (SM), ceramide (CER) and dihydroceramide (DCER), respectively. Data are mean ± SE of relative quantity (pg/µL). * indicate significance at *P* < .05

**Figure 9:**
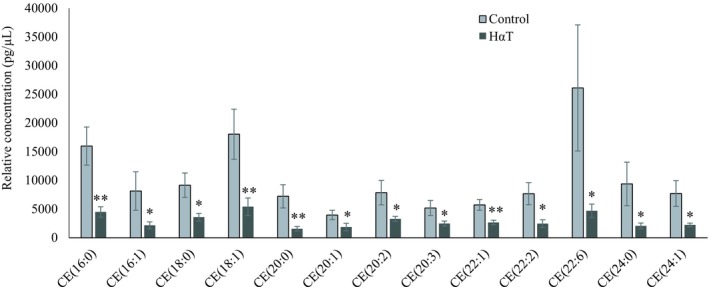
Relative quantity of cholesterol ester in freshly isolated urinary exosomes from HαT patients and healthy volunteers. Data are mean ± SE of relative quantity (pg/µL). * and ** indicate significant at 5% and 1% probability levels, respectively

Sphingolipids such as SMs and CERs play roles in modulating inflammatory signaling and response or in the secretion of exosomes into the extracellular milieu.^26^We compared levels of 12 lipids from each of 5 classes in this group, including SM, CER, DCER, HCER, LCER with a total number of 60 species scanned. Of this group, quantitation was obtained for all 12 SM, 12 CER and 12 DCER as well as for 7 HCER (Table 2). None of the LCER was quantified. Nineteen of the total (44%) were significantly altered. Among the identified sphingolipids, SM(18:1), SM(20:1), CER(20:0), CER(22:0), DCER(14:0), DCER(20:1) and DCER(22:1) were significantly reduced in HαT patients (Figure 8). Among the 21 different CE species identified, 13 CEs with 16, 18, 20, 22 and 24 carbons were significantly reduced in the urinary exosomes of HαT patients (Figure 9).

## 4 DISCUSSION

Exosomes and microvesicles are among the two main types of extracellular vesicles secreted by living cells.^33^ The lipid composition of microvesicles lacks the asymmetric distribution of lipids within the inner and outer leaflet of the plasma membrane. In general, exosomes are more biologically relevant in health and disease. For these reasons, we focused on the isolation of exosomes from the urine of HαT patients and healthy volunteers and the analysis of exosome lipids in this study. Importantly, all the HαT patients in our cohort had multiple alpha and beta copy numbers of the tryptase gene, high serum tryptase levels, and varying GI dysmotility and gut inflammation (Table 3). Since exosomes secretion and urine production may vary within a 24‐hour cycle, all urine samples were collected between 9‐11AM All patient and donor information were coded and not revealed until the data were analyzed at the end of the study.

Lipid profiles can vary among mast cell types that are derived from various tissue sources. Recent findings suggest that mast cells heterogeneity in humans is substantial, with morphology, receptor, enzyme, and mediator expression differing among various subtypes. For example, there is a more pronounced expression of 5‐lipoxygenase in mast cells located in the small airways and pulmonary vessels compared with mast cells in the central airways and parenchyma, leading to differential mast cell activation that depends on the stimulus and mast cell phenotype.^34^ Another study found that lipid metabolic profiles of two phenotypically distinct mast cell populations in mouse lung demonstrated differences in eicosanoid patterns of compounds derived mostly from arachidonic acid, which is activated by phospholipase A2 (PLA2), following stimulation with the conventional mast cell secretagogue A23187, a calcium ionophore.^35^

Our study is the first human study to profile lipids in urinary exosomes from HαT patients compared to healthy volunteers. Interestingly, here we show HαT patients excrete more exosomes in the urine compared to healthy volunteers. We chose to compare the lipid content within urinary exosomes between the HαT and control groups by normalizing to the number of exosomes, since exosomes isolated from healthy volunteers and HαT patients were approximately the same size, per volume of diluent that was used to resuspend the exosomes. This allowed us to make semi‐quantitative comparisons between lipid species within the two groups. Comparison of the quantified lipid species and significantly reduced species in HαT patients indicates that 64 out of 521 lipids species were lower in relative quantity in HαT patients relative to control volunteers. This means only 12% of the total identified lipids were significantly decreased in the urinary exosomes of the patients while roughly 88% of the lipids were similar in the two groups.

Skotland et al recently provided a comprehensive review of lipids in exosomes.^36^ In the current study, we used Splash internal standards but because there is not a standard for every lipid class, we can only provide relative quantitation. Among the lipids that were altered in HαT patients are phospholipids (PG, PI, PE and LPE), TAG, DAG, SM, and CER, all of which were reduced. The molecular composition of TAGs has been shown to be highly diverse and this group serves as an important free fatty acid resource and energy storage.^37^ The reduction observed suggests that HαT patients have less energy reserves than normal patients. Furthermore, reductions in bioactive molecules such as PI, cholesterol esters, SM, and CERs could be the basis for several of the comorbidities seen in HαT patients such as gastrointestinal disorders 38 and nervous system disorders.^39^ SM and CER have been linked to regulating inflammation and cell proliferation in these tissues. Cholesterol and SMs are the main constituents of signaling microdomains known as lipid rafts.^40^ Cholesterol has been shown to induce formation of lipid rafts while cholesterol depleting agents have been shown to lead to the breakdown of lipid rafts.^41^, ^42^ More research is necessary to identify the role of SM and CER in exosomes and how decreases in these lipids may serve as biomarkers of HαT.

In our study, urinary exosomes from HαT patients were found to have lower amounts of diacylglycerols and lysophosphatidylethanolamines compared to normal volunteers, implying that the activity of cellular phospholipase C and/or phospholipase A2 is lower in the HαT patients compared to the control volunteers. In addition, SMs and CERs are reduced in the HαT patient group, suggesting that the typical hydrolysis of SMs into phosphocholine and CER is altered in HαT patients. This lowered hydrolysis of SMs, which accounts for 5%‐10% of the phospholipids in plasma membranes of mammalian cells, could alter membrane fluidity and signal transduction. The lower DAG could also be associated with less hydrolysis of phosphatidylinositol 4,5‐bisphosphate (PIP2) and lower PLA/PLC2 activity. PIP2 has been associated with signaling to the proteinase‐activated receptor‐2 (PAR2) expressed on the surface of mast cells. The disparate oxylipin profiles found in the murine models of the Lundström study imply implications for disease pathology involving mast cells.^35^ Indeed, lipid mediator profiles were altered between lung compartments in asthmatics and healthy humans, with elevated oxylipins primarily from the lipoxygenase pathway of arachidonic and linoleic acid in asthmatics compared with healthy control volunteers, although the mast cells were not specifically implicated for these differences.^43^

All significantly reduced sphingolipids and most of the reduced phospholipids were saturated or mono‐unsaturated lipids. Consistent with our findings, others have also reported high abundance of saturated or mono‐saturated glycerophospholipids in the exosomes.^44^ Among all identified lipids, the relative quantity of PC was higher than other lipids. It has been shown that PC is the most abundant phospholipid that comprises cellular membranes including intracellular vesicles.^45^ PC is an important structural component of cellular membranes that is mainly synthesized by the Kennedy pathway and the methylation pathway.^45^ Sewell et al examined macrophage PC composition in Crohn's disease and found similar profiles between newly synthesized and endogenous PC species from healthy control and CD macrophages.^46^ Here, we examined, PC species within urinary exosomes from healthy volunteers and HαT patients. We observed that PC (18:1/18:1) and PC (18:2/16:1) were relatively lower in the HαT group compared to the healthy volunteer group.

Although exosomes that are excreted into the urine can come from various cell types, a large amount of these exosomes originates from the luminal side of polarized epithelial cells along the nephron. We thought high levels of prostaglandin D2 (PGD2) could be responsible for higher tubular exosome release, and a high level of PLD in HαT patients can be responsible for a greater production of exosomes from mast cells. It has been previously shown that PGD2 urine metabolites are increased in patients with mast cell disorders and these metabolites can be used as biomarkers.^47^, ^48^ In addition, PGE2 activates mast cell responses by inducing the mTORC2 pathway.^49^ Prostaglandin E2 and Prostaglandin I2 are known to increase renal blood flow and glomerular filtration rate via vasodilation.^50^ Thus, it is plausible to consider that prostaglandins may contribute to the increased excretion of exosomes in patients with HαT compared to the control healthy volunteer group. Phospholipase D plays an important role in mast cell activation.^51^ Previous studies have shown that Phospholipase D increases the release of exosomes.^52^

Several different exosome isolation procedures have been described by other groups.^53^ Size‐based isolation techniques such as ultrafiltration and ultracentrifugation methodologies have been optimized for efficient pore size and speed, respectively, in order to efficiently remove and prevent the break‐up of larger vesicles. This approach may result in the co‐isolation of aggregated lipids and proteins but is the most commonly used method because it offers the advantage of obtaining a high yield of isolated exosomes for subsequent lipidomic analyses. Immuno‐affinity capture‐based techniques such as the microplate‐based enzyme‐linked immunosorbent assay (ELISA) utilize an exosome biomarker antibody and offer a high throughput and potentially automated methods for screening samples for specific subsets of exosomes. However, these procedures rely on the specificity and sensitivity of an antibody and do not readily allow for a high enough yield of captured exosomes that is desired for lipidomics. Polymer‐based exosome precipitation procedures have been enabled without the need for special equipment. However, these approaches pose the problem of co‐immunoprecipitation of non‐exosome contaminants. Our exosome isolation procedure consisted of a series of filtration and differential centrifugation steps in which the pore size of the filter and centrifugal forces and durations were chosen for optimal exosome purity and yield.

Taken together, the results of our study reveal an upregulation in the secretion of exosomes and distinct differences in the lipidome of urinary exosomes isolated from HαT patients compared to healthy volunteers. There are several implications for various lipids that were found to be differentially enriched between the two groups. Since these lipids serve important structural and signaling roles, future studies are warranted to study these lipids in the context of disease mechanisms.

## CONFLICT OF INTEREST

There are no conflicts of interest to declare from any of the authors.

## AUTHOR CONTRIBUTIONS

SC Glover, MZ Nouri, KM Tuna, L. B. Mendoza Alvarez, LK Ryan, JF Shirley, Y. Tang, ND Denslow, and AA Alli designed the research, analyzed the data, wrote the paper, and approved the final version of the paper. MZ Nouri, KM Tuna, Y. Tang, and AA Alli performed the experiments.
